# IGF Binding Protein-5 Induces Cell Senescence

**DOI:** 10.3389/fendo.2018.00053

**Published:** 2018-02-20

**Authors:** Fumihiro Sanada, Yoshiaki Taniyama, Jun Muratsu, Rei Otsu, Hideo Shimizu, Hiromi Rakugi, Ryuichi Morishita

**Affiliations:** ^1^Department of Clinical Gene Therapy, Osaka University Graduate School of Medicine, Suita, Osaka, Japan; ^2^Department of Geriatric and General Medicine, Osaka University Graduate School of Medicine, Suita, Osaka, Japan

**Keywords:** IGF binding protein-5, cell senescence, inflammation, coagulation system, age-related disease

## Abstract

Cellular senescence is the complex process of deterioration that drives the aging of an organism, resulting in the progressive loss of organ function and eventually phenotypic aging. Senescent cells undergo irreversible growth arrest, usually by inducing telomere shortening. Alternatively, senescence may also occur prematurely in response to various stress stimuli, such as oxidative stress, DNA damage, or activated oncogenes. Recently, it has been shown that IGF binding protein-5 (IGFBP-5) with the induction of the tumor suppressor p53 is upregulated during cellular senescence. This mechanism mediates interleukin-6/gp130-induced premature senescence in human fibroblasts, irradiation-induced premature senescence in human endothelial cells (ECs), and replicative senescence in human ECs independent of insulin-like growth factor I (IGF-I) and IGF-II. Additionally, a link between IGFBP-5, hyper-coagulation, and inflammation, which occur with age, has been implicated. Thus, IGFBP-5 seems to play decisive roles in controlling cell senescence and cell inflammation. In this review, we describe the accumulating evidence for this role of IGFBP-5 including our new finding.

## Introduction

Insulin-like growth factor I (IGF-I) and II (IGF-II) are insulin superfamily members and are ubiquitously distributed in several organs (1, 2). Six high-affinity IGF binding proteins (IGFBPs) interact with IGFs, regulating IGF-I/II bioavailability, distribution, and signaling. IGFBPs are secreted and bind to IGFs in the circulation and extracellular environment (3). In addition to IGF-dependent action, IGF-independent functions of IGFBPs, many of which occur intracellularly, have recently been reported (4, 5). For example, IGFBP-1 and -2 are associated with cancer cell proliferation, adhesion, and migration through the specific binding of IGFBP-1 and -2 to alpha 5 beta1 integrin, followed by alterations in the phosphorylation status of downstream signaling molecules (6, 7). By regulating enzymes involved in sphingolipid metabolism, IGFBP-3 and -5 affect the balance between growth inhibitory lipids and growth stimulatory lipids (8, 9). Additional evidence has implicated that IGF binding protein-5 (IGFBP-5) is upregulation in the irradiation-induced premature senescence and replicative senescence of umbilical vein endothelial cells (HUVECs) (10). Knockdown of IGFBP-5 in aged HUVECs partially reversed the process of senescence, whereas the application of exogenous IGFBP-5 or IGFBP-5 overexpression induced premature senescence in HUVECs *in vitro* (11), indicating a decisive role for IGFBP-5 in controlling cell senescence and proliferation. The insulin/IGF signaling pathway has been implicated in the aging of many organisms, ranging from nematodes to mammals. The observation that IGFBPs modulate the availability or the distribution of IGF-1 adds further support to the hypothesis that IGFBPs have a vital role in the aging process (12). Many changes in the immune system, hemostasis, and vasculature, including alterations in inflammation, coagulation, and vascular senescence, occur during aging. However, its mechanism is not fully understood.

In this review, we first overview the mechanism of chronic inflammation during aging and later possible mechanism linking between cell senescence and senescence-inducing stimuli *via* IGFBP-5 is discussed.

## Mechanism of Chronic Inflammation in Aging

**Figure 1:**
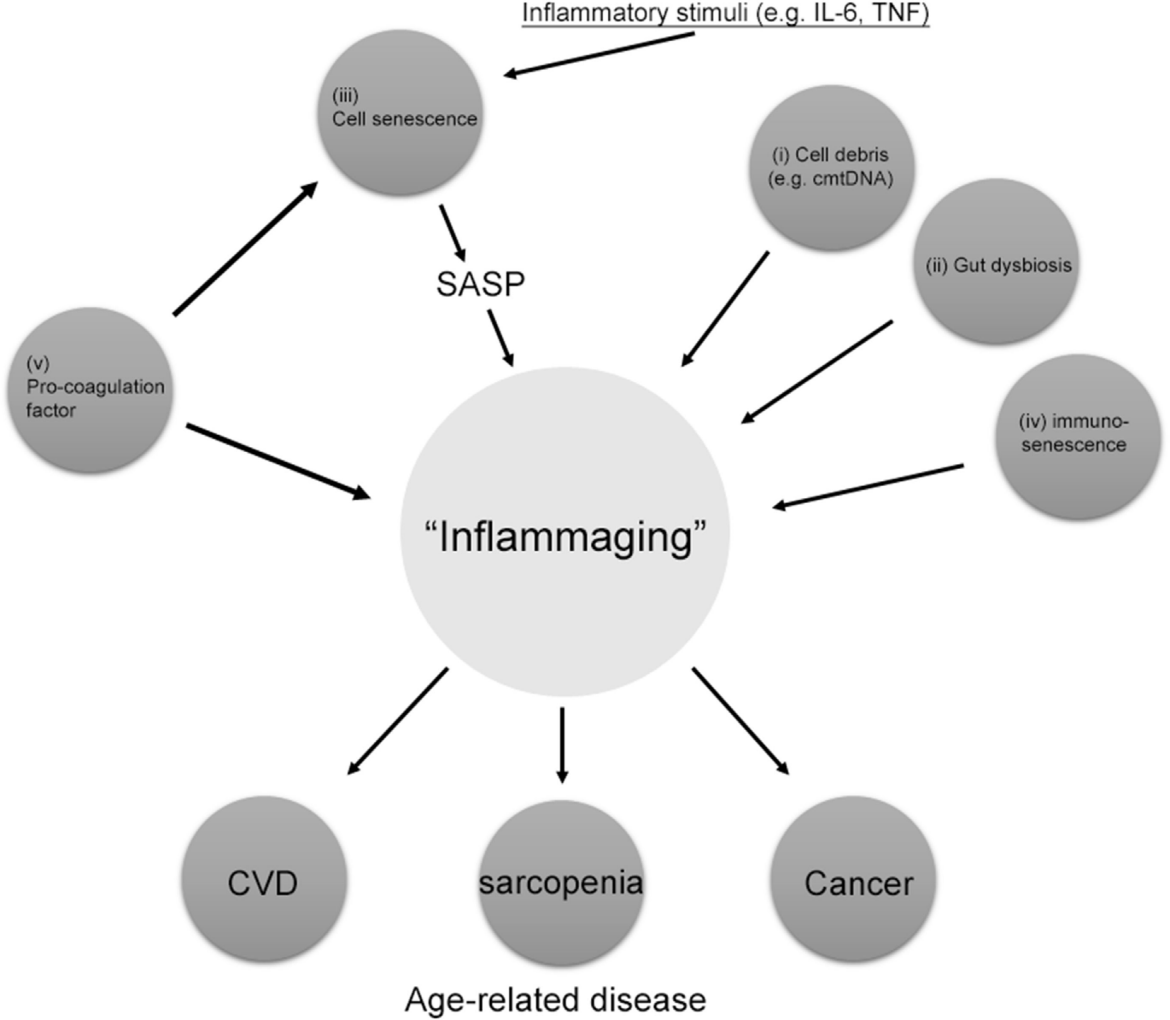
Source of “inflammaging.” Among the main causes of inflammaging, the accumulation of pro-coagulation factors, cell senescence, cell debris such as circulating mitochondrial DNA (cmtDNA), gut dysbiosis, and immune senescence are the main causes of inflammaging. Pro-coagulation factors also cause cell senescence. Inflammaging can also be influenced by many other factors, including age, reactive oxygen species, and those not directly related to inflammation, such as microRNAs (miRs) and agalactosylated *N*-glycans. SASP, senescence-associated secretory phenotype.

Inflammation is required for an acute, transient immune response to invading pathogens or acute traumatic injury. This process is essential for facilitating the tissue repair by increasing division and migration of cells. In contrast, chronic inflammation causes low-grade and persistent inflammation leading to tissue degeneration rather than the solution to infection, injury, or disease (13). Many aged tissues are chronically inflamed, which is the common pathological mechanism for age-associated diseases, such as cardiovascular disease, diabetes, cancer, and Alzheimer’s disease (14). Interleukin-6 (IL-6) and tumor necrosis factor-α (TNF-α) counteracts anabolic signaling, including insulin and erythropoietin cascades. Thus, chronic low-grade inflammation is now recognized as an important causative factor for insulin resistance and sarcopenia (15). Several sources of chronic inflammation during aging termed “inflammaging” have been described (Figure 1) (14). (i) Debris from cells or immunoglobulin accumulation due to increased cell death or inappropriate cell elimination systems in aging activates the innate immune system, resulting in chronic inflammation in aged organs (16). According to Zhang et al. (17), circulating mitochondrial damage-associated molecular patterns cause inflammation in response to injury. Mitochondrial damage-associated molecular patterns released from damaged cells are evolutionally conserved with bacterial pathogen-associated molecular patterns activating innate immunity (18). Thus, age-related failing of mitochondria quality control is associated with inflammaging. (ii) The ability of the oral and gut mucosa to protect against bacterial invasion declines with age, leading to persist low-grade inflammation (19). Periodontal disease has been also demonstrated to increase the inflammatory response with age (20). Additionally, the abundance of anti-inflammatory microbiota decreases with age and is inversely correlated with serum level of inflammatory cytokines, such as TNF-α and IL-1β (21). (iii) The increased number of senescent cells in tissue secretes various inflammatory cytokines, which play a causal role in age-related diseases. Senescent cells have been identified in age-related diseases including atherosclerosis, cancer, and diabetes (22–24). Senescence-associated secretory phenotype (SASP) is considered to be the main mechanism by which persistent prolonged inflammation occurs even after the initial stimulus has been removed. (iv) Age-related changes in the immune system termed “immunosenescence” increase the susceptibility to infections, malignancy, and autoimmunity, decrease the response to vaccinations, and impair wound healing (25, 26). These changes in the innate and adaptive immune responses associated with increasing age cause inappropriate inflammation. (v) Increased activity of the coagulation and fibrinolysis systems during aging has recently been reported to increase inflammation through the protease-activated receptors (PARs) (27, 28). The plasma concentrations of coagulation factors V, VII, VIII, and IX increase in healthy humans in parallel with the physiological processes of aging. In addition, fibrinogen levels, which have emerged in several trials as a primary risk factor for thrombotic disorders, have been shown to increase with advancing age (29). Thus, uncontrolled coagulation activity and the subsequent activation of the fibrinolysis system contribute to the pathophysiology of aging and several age-related chronic inflammatory diseases, such as atherosclerosis and lung fibrosis (30).

## IGFBP-5 and Cell Senescence

Increasing evidence has implied that the clearance of senescent cells in animal models attenuates the progression of age-related disorders, including osteoarthritis and atherosclerosis (31–33). This evidence strongly supports the hypothesis that senescent cell clearance or the modulation of pro-inflammatory pathways related to the acquisition of SASP might be potential therapeutic strategies for combating age-related diseases and expanding the health span of humans. The IGF/IGFBPs system has been implicated to be a potential target of age-related disease. Of the six IGFBPs, IGFBP-5 plays a critical role in the process of replicative and premature cell senescence (10, 11). PAR-1/2 signaling induced by coagulation factor Xa (FXa) and the fibrinolytic factor plasmin has been shown to increase IGFBP-5 expression in endothelial cells (ECs) and smooth muscle cells (SMCs) (34–36). FXa stimulation of ECs and SMCs increased inflammatory cytokine secretion *via* enhancing cellular senescence through the early growth response 1–IGFBP-5–p53 pathway (34, 37). Interestingly, the FXa- and IGFBP-5-positive areas within the atherosclerotic plaques of human were similarly distributed (37). Kojima et al. have demonstrated that IGFBP-5, as a major signal transducer and activator of transcription 3 mediator, regulates IL-6-induced reactive oxygen species (ROS) production, as well as the subsequent DNA damage response and senescence of TIG3 fibroblast cells (38). They also discovered that IGFBP-5 itself had senescence-inducing activity in TIG cells with increased ROS production. Knocking down of IGFBP-5 significantly reduced IL-6/IL-6R-induced ROS increase and premature senescence. Together, all of these data support the hypothesis that IGFBP-5, which is produced in p53-dependent manner, plays an important role in FXa- or IL-6-induced premature senescence of ECs, SMCs, and fibroblast. IGFBP-5 plays a multifunctional role, possessing growth inhibitory and growth promoting functions (39). IGFBP-5 in breast cancer cells enhances cell proliferation (40). In contrast, IGFBP-5 transgenic mice show retarded growth and reduced litter size (41). Additionally, IGFBP-5 directly regulates apoptosis by interfering with the IGF signaling cascade (42). Moreover, cytoplasmic accumulation of IGFBP-5 in breast cancer cells interacted with sphingosine kinase and protein kinase C, stimulating antiapoptotic effects (9, 43). Thus, IGFBP-5 seems to exert its multifunction depending on cell type, pattern of its distribution in cells and tissue, and IGF-I/II bioavailability.

IGFBP-3, -4, and -6 are also associated with the process of cell senescence. Through Akt, p53, FOXO3a, IGFBP-3 promotes ECs and fibroblast senescence (44, 45). Senescent mesenchymal stem cells secrete IGFBP-4, and it promotes their senescence (39). Senescence induced by pro-oxidative stimuli increases IGFBP-6 levels and IGFBP-6 enhances cell senescence in fibroblast (46, 47), although some experiments demonstrated contradictory results (48). Structurally, six IGFBPs have highly conserved N- and C-terminal domains (49) and different protein sequence in the linker domains (3). Considering their tissue distribution pattern (50), the six IGFBPs might have similar effect on cell senescence in different tissues. Alternatively diverse posttranslational modification in the linker domain of IGFBPs during aging might modify their function on cell growth and senescence. Additional work is required for the elucidation of their function in cell senescence.

## Conclusion

**Figure 2:**
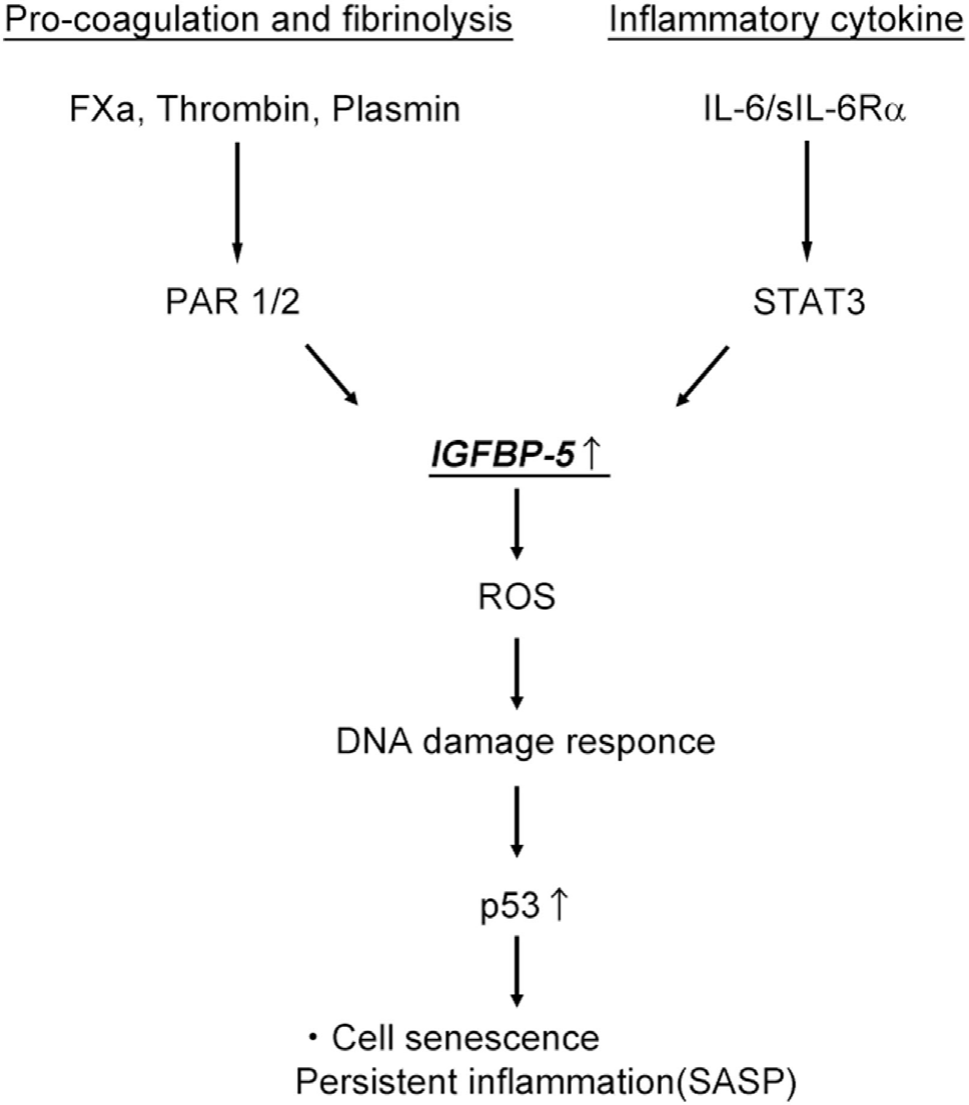
Activation of the pro-coagulation cascade and interleukin-6 (IL-6)/signal transducer and activator of transcription (STAT3) pathway induce cell senescence and persistent inflammation through IGF binding protein-5 (IGFBP-5). ROS, reactive oxygen species; SASP, senescence-associated secretory phenotype.

IGF binding protein-5 has decisive roles in controlling cell senescence and subsequent cell inflammation independent of IGF-I and -II. IGFBP-5 expression was recently shown to be increased following stimulation with coagulation fXa, plasmin, IL-6, and irradiation, leading to cell senescence (Figure 2). Additionally, IGFBP-5 induces fibroblast activation and the inflammatory response, contributing to tissue fibrosis. Currently, information on the roles of IGFBPs in the aging of different cells and tissues and the molecules related to IGFBPs signaling is limited. Therefore, the molecular mechanisms underlying the effect of the IGFBP system on aging requires further research. Therapies targeting the coagulation and fibrinolysis cascades might represent new options for the treatment of chronic inflammatory diseases.

## Author Contributions

FS and YT organized, performed experiment, and wrote manuscript. JM, RO, and HS collected data. HR and RM supervised experiment.

## Conflict of Interest Statement

RM received research funding from Bayer Yakuhin, Ltd. Other authors have no conflicts of interests.
